# Laryngeal Cavernous Lymphangioma in an Adult Patient

**DOI:** 10.22038/IJORL.2021.55065.2889

**Published:** 2021-11

**Authors:** Recep Bedir, Muhammet Safa-Ayazoğlu, Metin Çeliker

**Affiliations:** 1 *Department of Pathology* *, Recep Tayyip Erdoğan University School of Medicine, * *Rize, Turkey.*; 2 *Department of Otorhinolaryngology, Recep Tayyip Erdogan University School of Medicine, Rize, Turkey.*

**Keywords:** Adult, Lymphangioma, Larynx

## Abstract

**Introduction:**

Lymphangiomas are uncommon congenital lesions of the lymphatic system, and most of them are detected by the second year of life. Although head and neck region is the most common location, laryngeal involvement isextremely rare, and the literature is largely limited to few case reports.

**Case Report:**

A 51-year-old male patient was admitted to the hospital with clinical history of hoarseness. Performed direct laryngoscopy revealed a pedunculated cystic mass located in the right ventricle of the larynx. The mass was totally excised, and the case was reported as cavernous lymphangioma.

**Conclusion:**

Isolated laryngeal lymphangioma is extremely rare in adults. The other benign lesions or inflammatory processes in this region can mimic laryngeal lymphangioma. Therefore, this entity should be kept in mind in differential diagnosis especially in adults, to avoid overtreatment.

## Introduction

Lymphangiomas are rare, congenital neoplasms of lymphatic system, seen early in childhood. Despite mostly arising in the head and neck region, laryngeal involvement is extremely rare. Laryngeal lymphangioma constitutes 50-60% of all lymphatic malformations in the newborn, as well as 80-90% of children under 2 years of age, whereas is extremely rare in adults (1,2). 

Surgical excision is the main treatment method, however sclerotherapy can be performed when surgery is not available. Here, we present an unusual case of laryngeal cavernous lymphangioma in an adult patient.

## Case Report

**Fig 1:**
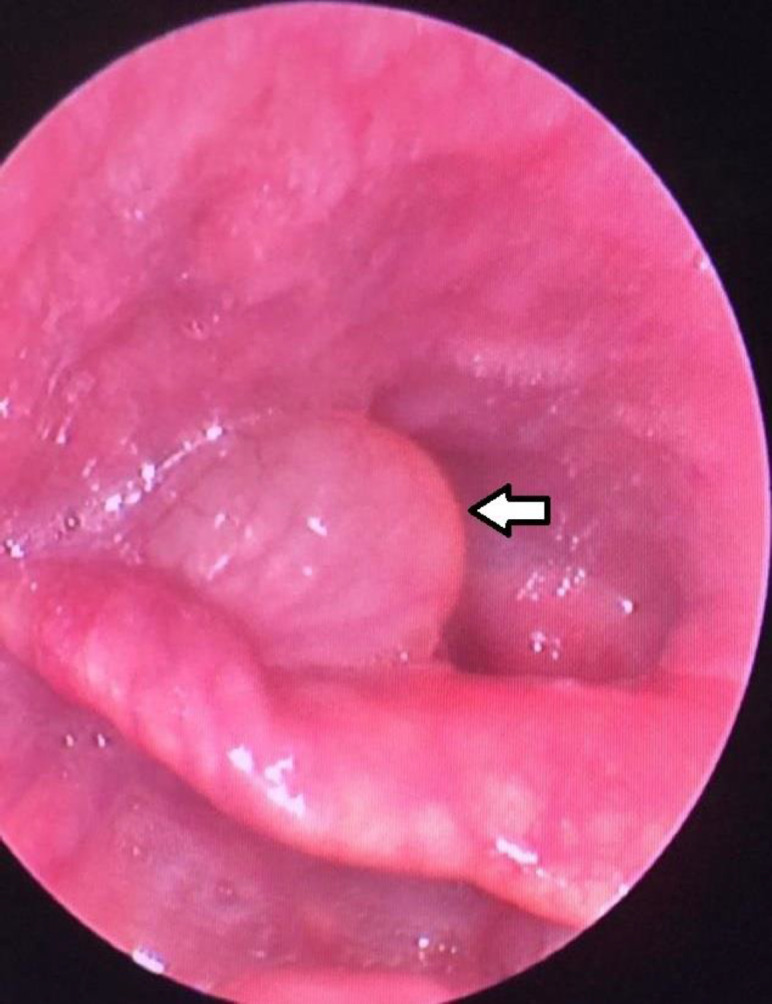
Direct laryngoscopy reveals a gray-red cystic lesion in the right ventricle

A 51-year-old-male patient was admitted to the hospital with clinical history of hoarseness for three months. Performed direct laryngoscopy revealed a gray-red cystic lesion with a diameter of 0.6 cm, located at the right ventricle (Fig 1). 

**Fig 2:**
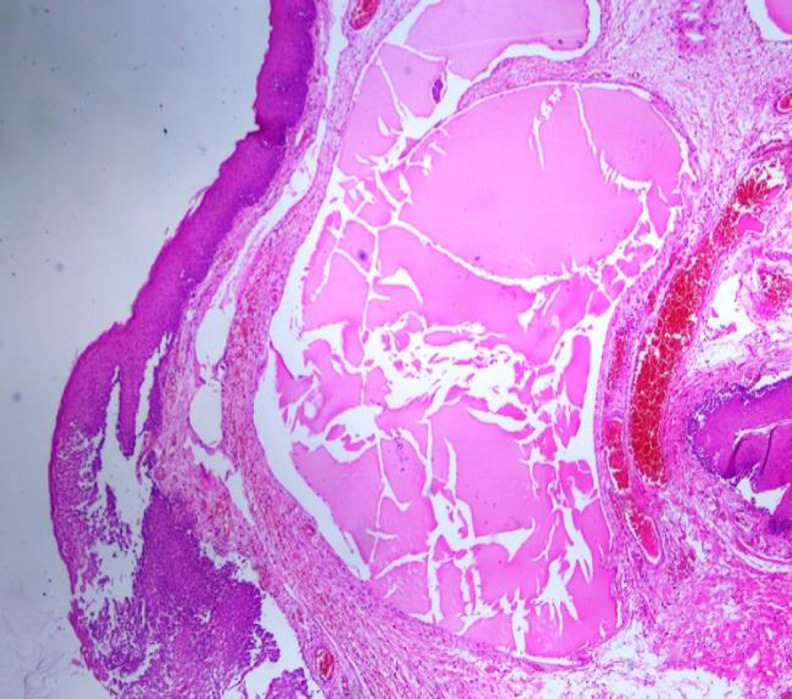
Microscopic appearance of the lymphangioma under the stratified squamous epithelium (HE, X200)

Moreover,there was a thyroglossal cyst, 3x2 cm in size, located at the midline of the neck**.** The lesion was excised, and histopathological examination revealed enlarged lymphatic vessels with content of eosinophilic, proteinaceous material (Fig 2). 

**Fig 3:**
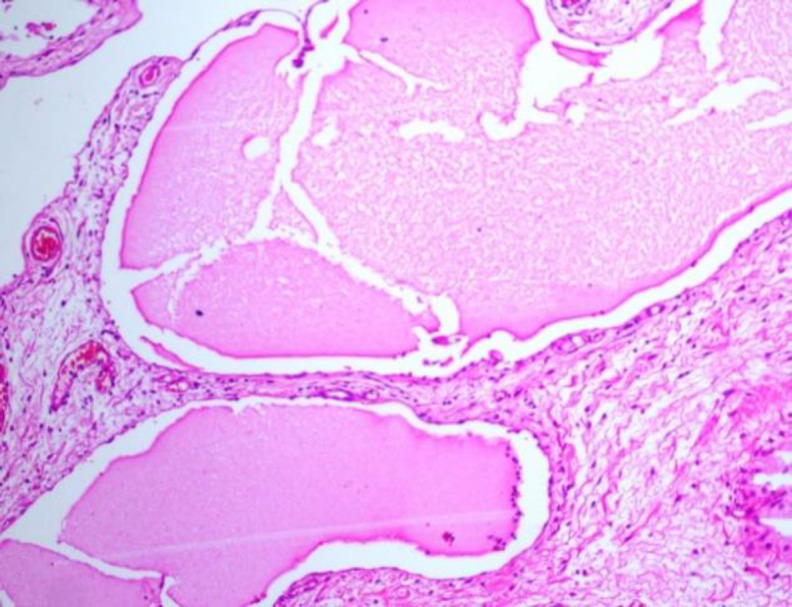
Lymphangioma composed of ovoid dilated channels, some of which contains eosinophilic, proteinaceous material (HE, X200)

**Fig 4:**
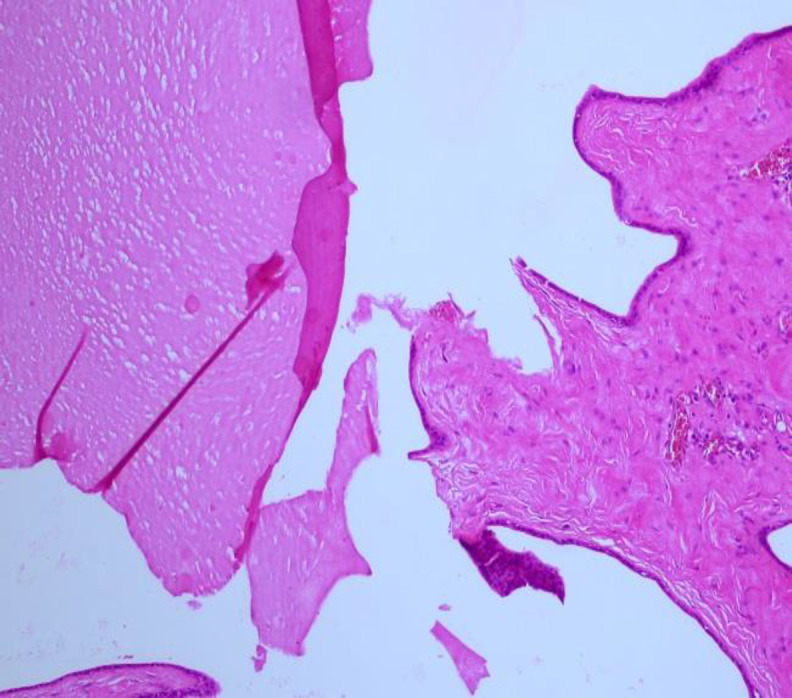
Dilated lymphatic channels lined with flattened endothelium (HE, X400)

The wall of lymphatic vessels had flattened endothelium (Fig 3,4). 

**Fig 5:**
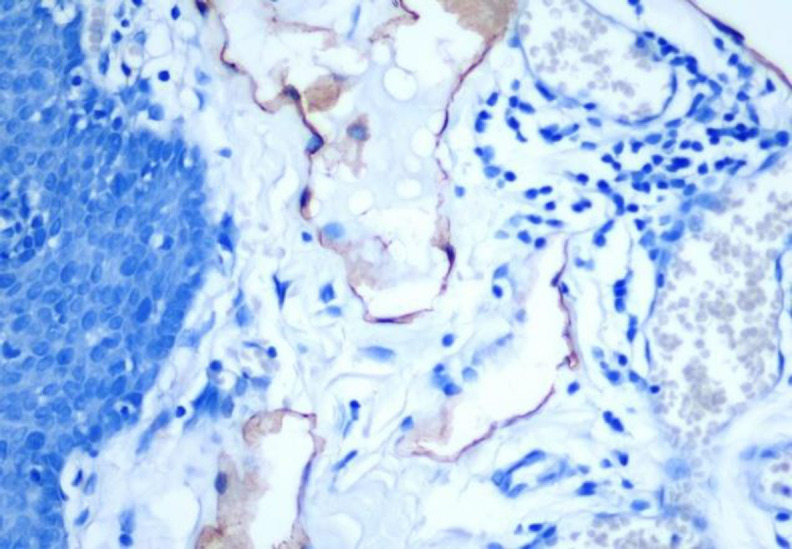
The flattened endothelium was positive for D2-40 (X400)

Performed immunohistochemical staining revealed that endothelial lining was positive for CD31 and D2-40 (Fig 5). 

**Fig 6:**
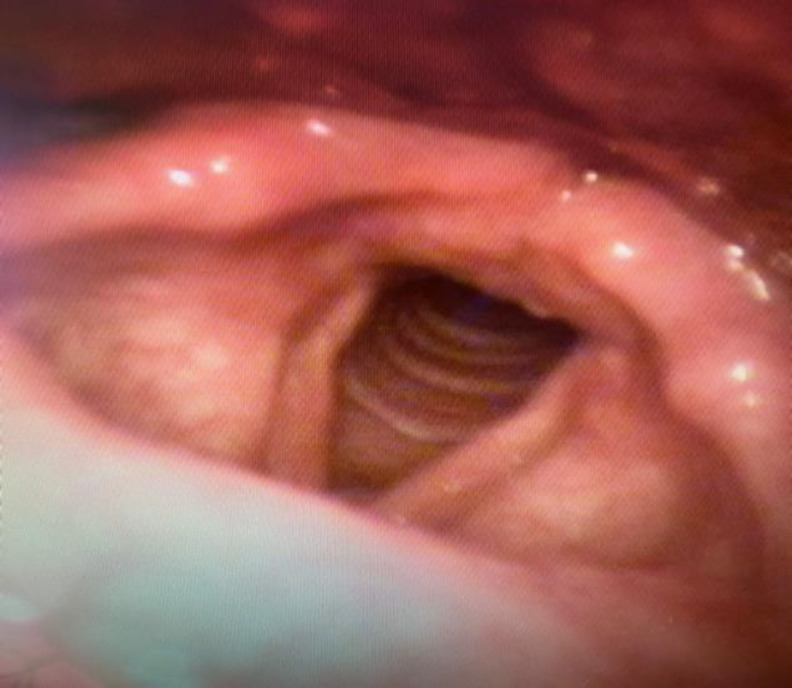
Postoperative performed direct laryngoscopy reveals no space-occupying lesion

The case was reported as laryngeal cavernous lymphangioma. The patient has been followed up regularly by performing direct laryngoscopy. No local recurrence has been detected during the one-year follow-up (Fig 6).

## Discussion

Lymphangiomas are rare congenital benign lesions, mostly arisen in the head and neck region. Laryngeal involvement is extremely rare, and the literature is largely limited to few case reports (1-6).

They are generally in the form of extension of congenital neck lymphangiomas or can be associated with cystic hygroma. Even mostly they are seen in infants, some may present in young adults and adults. For example, Seven et al, Gupta et al and Stankovic et al reported isolated laryngeal lymphangiomas of a 37-year-old male, 13-year-old girl and 42-year-old male respectively (3,10,11). Our case who was a 51-year-old male patient was unusual with being not only adult, but also having older age than reported cases in literature.

Mostly, laryngeal lymphangioma arises in the epiglottis, aryepiglottic folds, band ventricle or arytenoids. Most common presenting symptoms are voice change, snoring, dysphagia, and difficulty in swallowing. Moreover, symptoms can rapidly worsen when the lesion reach a larger size, and sudden dyspnea can be seen due to airway obstruction (11). The symptoms can mimic other benign lesions or inflammatory processes which cause misdiagnosis, especially in adults. Therefore, awareness of this entity is crucial to make a definite diagnosis and avoid overtreatment (3,4,6). Radiological findings play an important role in diagnosis, however, some benign lesions such as hemangiomas can mimic lymphangiomas.

Thus, definite diagnosis can only be made by histopathologic examination (10). In our case, hemangioma was in our differential diagnosis, but the presence of eosinophilic proteinaceous material within the lymphatic vessels excluded the possibility of hemangioma. Moreover, hemangiomas and lymphangiomas have similar immunohistochemical findings. In our case, endothelial lining was positive for CD31, CD34 and Factor VIII. These findings can also be seen in hemangiomas (5). However, additional D2-40 positivity supported our diagnosis of lymphangioma.

Lymphangiomas are divided into four subtypes by Kennedy as follows: superficial cutaneous, cavernous, cystic hygroma, and diffuse systemic (7). 

Cavernous lymphangiomas consist of dilated lymphatic channels with flattened or double layered endothelial layers, which are known as cystic hygroma in neonates. In our case, the subtype was cavernous, and the patient had concurrent thyroglossal cyst instead of cystic hygroma.

Despite spontaneous resolution of laryngeal lymphangioma can be observed, sclerotherapy and surgical excision are still the main treatment methods for lymphangiomas. Sclerotherapy is preferred if the lesion is small, since it is important to keep the vital structures around. However, this method has some disadvantages, since sclerosing agent can cause inflammatory reactions or damage adjacent structures (8,10).

Lymphangiomas have recurrence rates from 39% to 50% in patients following treatment (9).In our patient, the lesion was able to be excised, and no recurrence has been observed during the 1-year follow-up after excision.

## Conclusion

***We reported this case, since isolated laryngeal lymphangioma is extremely rare in adults. Moreover, preoperative diagnosis can be difficult, since inflammatory conditions and other benign lesions can mimic lymphangiomas. Mostly, surgical excision is preferred, since sclerotherapy can cause complications, as well as damage adjacent structures. Although rare in occurrence in adults, awareness of this entity is crucial for definite diagnosis and to avoid overtreatment.***
